# Association between intrahospital transfer and hospital-acquired infection in the elderly: a retrospective case–control study in a UK hospital network

**DOI:** 10.1136/bmjqs-2020-012124

**Published:** 2021-01-25

**Authors:** Emanuela Estera Boncea, Paul Expert, Kate Honeyford, Anne Kinderlerer, Colin Mitchell, Graham S Cooke, Luca Mercuri, Céire E Costelloe

**Affiliations:** 1 Global Digital Health Unit, Department of Primary Care and Public Health, Imperial College London, London, UK; 2 Department of Mathematics, Imperial College London, London, UK; 3 Tokyo Tech World Research Hub Initiative, Tokyo Institute of Technology, Tokyo, Japan; 4 St Mary’s Hospital, Imperial College Healthcare NHS Trust, London, UK; 5 Infectious Diseases Section, Imperial College London, London, UK; 6 Information Communications and Technology Department, Imperial College Healthcare NHS Trust, London, UK

**Keywords:** nosocomial infections, transitions in care, health services research

## Abstract

**Background:**

Intrahospital transfers have become more common as hospital staff balance patient needs with bed availability. However, this may leave patients more vulnerable to potential pathogen transmission routes via increased exposure to contaminated surfaces and contacts with individuals.

**Objective:**

This study aimed to quantify the association between the number of intrahospital transfers undergone during a hospital spell and the development of a hospital-acquired infection (HAI).

**Methods:**

A retrospective case–control study was conducted using data extracted from electronic health records and microbiology cultures of non-elective, medical admissions to a large urban hospital network which consists of three hospital sites between 2015 and 2018 (n=24 240). As elderly patients comprise a large proportion of hospital users and are a high-risk population for HAIs, the analysis focused on those aged 65 years or over. Logistic regression was conducted to obtain the OR for developing an HAI as a function of intrahospital transfers until onset of HAI for cases, or hospital discharge for controls, while controlling for age, gender, time at risk, Elixhauser comorbidities, hospital site of admission, specialty of the dominant healthcare professional providing care, intensive care admission, total number of procedures and discharge destination.

**Results:**

Of the 24 240 spells, 2877 cases were included in the analysis. 72.2% of spells contained at least one intrahospital transfer. On multivariable analysis, each additional intrahospital transfer increased the odds of acquiring an HAI by 9% (OR=1.09; 95% CI 1.05 to 1.13).

**Conclusion:**

Intrahospital transfers are associated with increased odds of developing an HAI. Strategies for minimising intrahospital transfers should be considered, and further research is needed to identify unnecessary transfers. Their reduction may diminish spread of contagious pathogens in the hospital environment.

## Introduction

In recent years, pressures from an ageing population coupled with inpatient bed reductions^1^ have highlighted the importance of optimising patient flow, which encompasses patient transfers between hospitals (interhospital) and within hospitals (intrahospital). Intrahospital transfers have been variously defined in the literature as any change of the patient’s location within the hospital, including transfers between the emergency department (ED) and an inpatient ward, a ward and a procedure room, or two beds on the same ward.^2^ Clinical factors such as the need for a procedure or isolation due to infection may require transferring the patient.^3^ However, minimal bed availability can also induce extra intrahospital transfers.^4^ Strategies such as using empty beds in short stay units for patients who are likely to need a longer term admission,^4 5^ or temporarily admitting patients as ‘outliers’ to clinically inappropriate wards with available beds,^6^ have been used to prevent ED congestion and meet the ‘4-hour rule’, which stipulates that patients in ED should be assessed within 4 hours of admission. A direct consequence of such strategies is that patients incur extra intrahospital transfers, with elderly patient populations disproportionately affected.^7 8^

Intrahospital transfers have been linked to a number of adverse events such as increased falls, length of stay, medication errors, delirium and hospital-acquired infections (HAIs).^2^ However, despite being established as an avenue for transmission of pathogens between hospitals,^9–11^ there is still a lack of clarity around the relationship between intrahospital patient movement and the risk of HAI.^2 12^ HAIs are defined as infections which have developed in a hospital or other healthcare delivery setting 48 hours or more following admission, or prior to this in a patient discharged in the preceding 48 hours.^13^ They place a significant burden on health systems, leading to increased mortality, intensive care unit (ICU) admissions and longer hospital spells.^14^ Several factors could underlie a possible association between HAIs and intrahospital transfers. Patients with frequent transfers are exposed to a greater number of environments and contacts in the hospital, increasing their risk of exposure to pathogens from contaminated surfaces, other patients or healthcare workers.^15–18^ Transferred patients may also experience delays in receiving care on admission to a new unit, thus extending their hospital stay,^3 19^ which is itself a risk factor for HAIs.^20^

While some cross-sectional studies have explored the association between HAIs and intrahospital transfers, these are subject to reverse causality bias, as any transfers following HAI diagnosis cannot be implicated in its cause.^19 21 22^ We identified only one case–control study considering room transfers prior to infection. The group reported that for each additional room transfer, the odds of becoming infected with *Clostridium difficile* infection (CDI) increased by 7%,^15^ and showed that cases have a more dispersed hospital coverage than controls using network analysis.^23^ Bush *et al* also showed that units with high incoming transfer rates were statistically associated with new cases of CDI.^12^

To the best of our knowledge, analyses of intrahospital transfers have not yet been linked with a broad range of microbiology data, despite the fact that many other nosocomial pathogens can contaminate hospital surfaces.^24 25^ Moreover, no such analyses have been conducted using UK healthcare system data, or information from multiple hospital sites. The present study applies statistical modelling to explore how the number of intrahospital transfers patients undergo influences the odds of developing any HAI in an urban hospital network. The network consists of five hospitals across four sites which together comprise a hospital ‘trust’. As elderly patients make up a large proportion of hospital inpatients, and HAIs are more prevalent in this population, the analysis is focused on those over the age of 65.^26^

## Methods

A retrospective case–control study was conducted using routinely collected electronic health records (EHR) and microbiology data.

### Data sources

Individual-level data of patients admitted during a 3-year period falling between January 2015 and December 2018 to the hospital trust were extracted from the electronic system used to routinely record patient health information. The data were anonymised, and accessed in a secure environment. The data have a hierarchical structure; an anonymised unique ID is given to each patient, and a unique ID to each hospital spell of a patient, which begins when the patient is admitted and ends due to death or hospital discharge.^27^ Each hospital spell can contain multiple consultant ‘episodes’, which signify a change of the consultant responsible for the patient’s care. The original EHR data set provided 53 variables including: timestamped day of admission, discharge, ward IDs with entry and exit times, and treatment function codes (TFC), which refer to areas of clinical work based on the main specialty or subspecialty of the healthcare professional responsible for the patient.^28^ In addition, diagnoses (International Statistical Classification of Diseases and Related Health Problems 10th Revision (ICD-10)) and procedures (Office of Population Censuses and Surveys Classification of Interventions and Procedures (OPCS-4)) aggregated by episode of care were included.^29^ The data set was linked to a diagnostic microbiology data set by hospital spell ID, which provided the sample collection time and date, site and the causative organism if present.

### Hospital trust characteristics

Of the five hospital sites, two were excluded from the analysis as they pertain to specialist maternity and ophthalmology centres. The remaining three sites contain approximately 1130 acute beds, including 136 critical care beds. Wards contain between 7 and 25 beds, with most wards comprising four to six bedded bays, while a small minority of wards contain only single rooms.

### Patient population

The patient sample was restricted to non-elective patients, aged 65 and over, who had a hospital spell duration of at least 48 hours. Non-elective spells, which refer to unscheduled hospital admissions, were selected in order to create a more uniform patient population with regard to inpatient movement. In addition, patients categorised under a surgical TFC were excluded (online supplemental table 1), as surgical patients experience a higher number of intrahospital transfers, are likely to be prescribed antibiotics prophylactically and have a heightened risk of infection.^30^ Patients who developed an infection in the first 48 hours after admission were also excluded from the analysis, regardless of whether they had had a recent admission, in order to remove all likely cases of community-acquired infections. A flow chart of the full exclusion criteria of study participants is provided (online supplemental figure 1).

10.1136/bmjqs-2020-012124.supp1 Supplementary data

### Outcome measure

The primary outcome was diagnosis of an HAI, defined as a positive laboratory culture (or PCR and/or immunoassay for CDI diagnosis, as per the European point prevalence survey definition, see online supplemental table 2)^31^ collected at least 48 hours after hospitalisation. The sample collection date was calculated as days and fractions of days based on the time and date the sample was taken. This was used to define the time of HAI diagnosis. If patients developed more than one infection during their hospital spell, the sample collection time and organism of their first infection was selected. Only one organism was recorded per sample, therefore coinfections were beyond the scope of this study.

### Intrahospital transfers

The main exposure of interest was number of intrahospital transfers undergone during time at risk. We define time at risk for each spell as the length of time between a patient’s admission and the first positive culture for cases, and discharge or death for controls. All time-dependent covariates were computed for the duration of time at risk in days and fractions of days, based on the timestamp of the variable (see Covariates section). Changes in ward ID were used to derive the number of intrahospital transfers undertaken. Any change in ward ID was considered an intrahospital transfer, irrespective of the time spent in the new location, therefore capturing temporary transfers to procedure rooms or admissions and discharge lounges. Bed transfers on the same ward were not included as these transitions are not timestamped, but bed transfers are highly correlated with the intrahospital transfer count used (online supplemental text 1). In addition, as the data set started from inpatient admission, transfers from the ED to the first inpatient ward were not included, while all intrahospital transfers that occurred between in-hospital wards, including ambulatory emergency care centres, were used in the transfer count.

### Covariates

**Figure 1:**
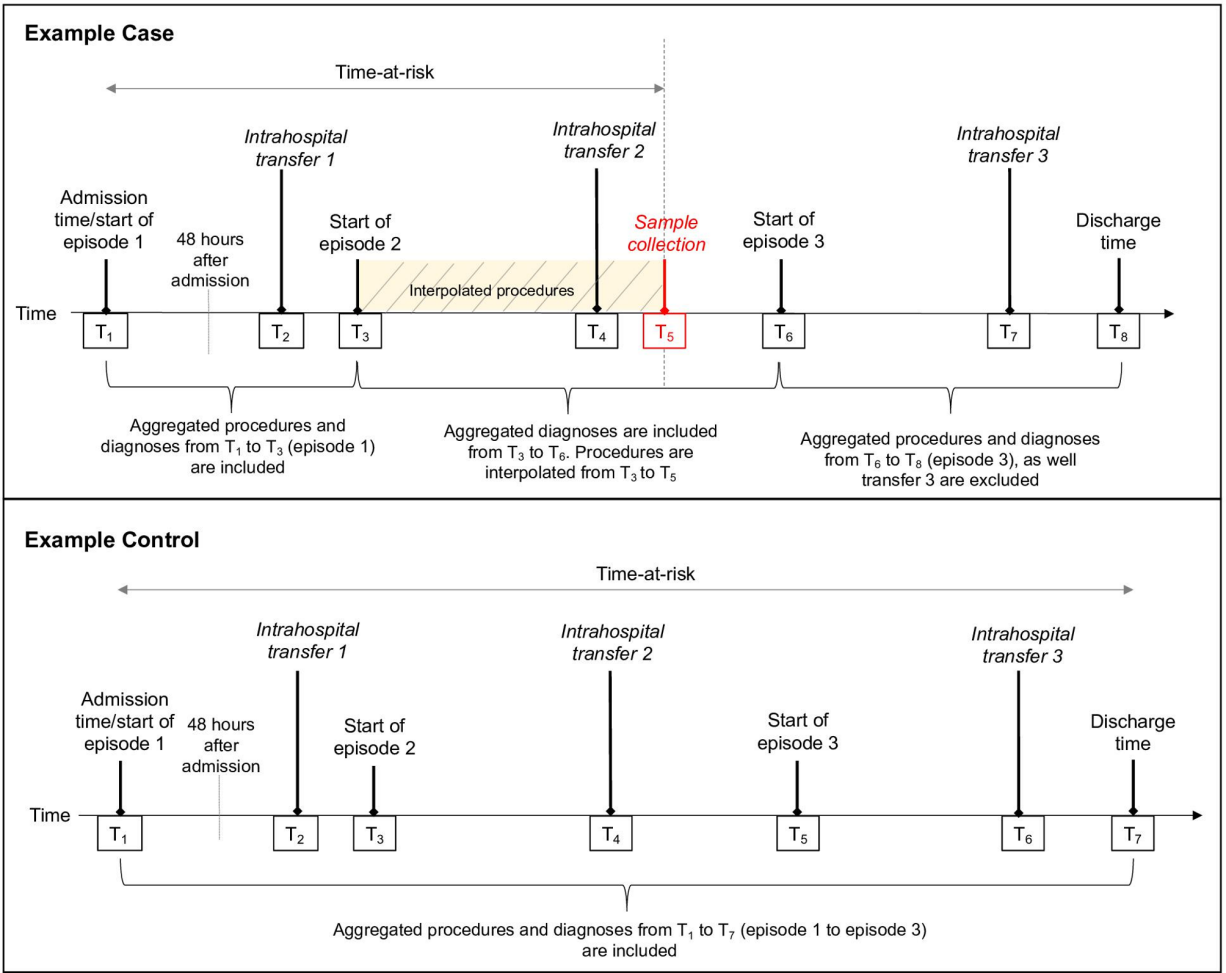
Illustration of time at risk definition, and time stamping in the EHR dataset using a fictitious case and control. Time at risk, intrahospital transfers and TFC were continuously monitored, giving a precise timestamp for their occurrence, while OPCS-4 and ICD-10 codes which are aggregated within consultant episodes. Although the optimal method of counting the OPCS-4 and ICD-10 codes for cases is to only include those which occurred from T_1_ to T_5_, due to the resolution of time stamps available in the data, only those from T_1_ to T_6_ were available. The procedure number in such episodes was interpolated between T_1_ to T_5_.

Elixhauser comorbidities, which comprise 31 comorbidity indicators, based on ICD-10 codes present in the episodes of care which began before the collection date were used to create a composite comorbidity measure per hospital spell, to provide comorbidity risk adjustment.^32^ Procedures were also included in risk adjustment because specific intrahospital transfers may be associated with heightened risk of infection due to the nature of the procedures that occur in the new location. For example, contaminated endoscopes and haemodialysis machines have been shown to be silent reservoirs for HAIs,^33 34^ therefore, transfers to dialysis and endoscopy procedure locations would appear to have an increased risk. OPCS-4 codes include a broad range of procedures for the diagnosis and treatment of disease, ranging from complex operations, such as transplants, to minor incisions, and non-operative procedures such as medical imaging.^29^ No classification system exists for OPCS-4 codes, therefore these were enumerated per hospital spell. All OPCS-4 codes were included from the episodes of care that began and ended *before* the sample collection date. However, for the episodes of care containing the collection date, a linear interpolation was conducted to estimate the number of procedures only up to collection date, assuming that procedures were evenly distributed over the duration of the episode (figure 1).

Interpolated procedure number was computed by:

$$\frac{\left(Sample\;Collection\;Date-Episode\;Start\;Date\right)}{\left(Episode\;End\;Date-Episode\;Start\;Date\right)}\times Total\;procedures\;during\;infection\;episode$$

The number of procedures in this episode was interpolated independently to the episodes which ended before the collection date and the two were combined to obtain the final procedure number. While the principal diagnosis of patients was not available from the EHR data, the patient’s TFC up to collection date for cases, or hospital exit for controls, was used to categorise individuals into 20 disease-related groups, and control for differences between patient groups. If multiple TFCs were assigned over a patient’s spell, the TFC under which the patient spent the longest duration was taken. Patient discharge location was also included as a categorical covariate, as discharge to a nursing home, another hospital provider or death is indicative of a frailer patient than those discharged to their own residence. Discharge destination has also been previously associated with increased ward transfers.^19^ Lastly, as patients residing in ICUs have a higher likelihood of developing an HAI,^35^ ICU admission before collection date for cases, or hospital exit for controls was included as a covariate. The final model was adjusted for age, gender, time at risk, Elixhauser comorbidities, hospital site of admission, dominant TFC, ICU admission, total number of procedures and discharge destination.

### Statistical methods

The unit of analysis in the study is the individual hospital spell. Data were initially explored descriptively to ascertain the baseline characteristics of hospital spells. Covariates which displayed a non-linear relationship with the outcome variable were grouped into categories with a similar relation to the outcome. A table of the categories chosen and numbers of observations in each category is given (table 1). Comparisons between cases and controls were conducted using χ^2^ tests.

**Table 1:** Characteristics of the 24 240 hospital spells, stratified by cases and controls. In addition, the frequency and percentage of patients across the categories of covariates used in the multivariable regression are given, with corresponding χ^2^ tests for significance (see online supplemental material for full table)

Characteristic	All spells (n=24 240)		Controls (n=21 363)		Cases (n=2877)		P value
	n	%	n	%	n	%	
Gender							
Male	12 032	49.64	10 592	49.58	1440	50.05	0.635
Female	12 208	50.36	10 771	50.42	1437	49.95	
Age							
Median, IQR	79	72–86	79	72–85	79	73–86	
65–70	4740	19.55	4248	19.88	492	17.10	0.004
71–75	4155	17.14	3666	17.16	489	17.00	
76–80	4723	19.48	4144	19.4	579	20.13	
81–85	4516	18.63	3977	18.62	539	18.73	
86+	6106	25.19	5328	24.94	778	27.04	
Attended ICU							
No	23 642	97.53	20 958	98.1	2684	93.29	<0.001
Yes	598	2.47	405	1.90	193	6.71	
Elixhauser comorbidities							
Mean, SD	3.54	1.9	3.48	1.89	4.00	1.98	
0	695	2.87	651	3.05	44	1.53	<0.001
1–3	12 265	50.6	11 061	51.78	1204	41.85	
4–6	9516	39.26	8204	38.4	1312	45.6	
7–9	1685	6.95	1386	6.49	299	10.39	
10 or more	79	0.33	61	0.29	18	0.63	
Time at risk (days)							
Median, IQR	6.30	3.61–11.74	6.31	3.60–11.72	6.21	3.69–11.85	
2–5	9756	40.25	8610	40.3	1146	39.83	0.016
5–7	3614	14.91	3154	14.76	460	15.99	
7–10	3501	14.44	3115	14.58	386	13.42	
10–15	3270	13.49	2890	13.53	380	13.21	
15–20	1634	6.74	1433	6.71	201	6.99	
20–30	1480	6.11	1275	5.97	205	7.13	
30–40	611	2.52	541	2.53	70	2.43	
40+	374	1.54	345	1.61	29	1.01	
Procedures							
Median, IQR	2	0–5	2	0–5	2	0–5	
Procedures (n)	7866	32.45	7057	33.03	809	28.12	<0.001
1	1854	7.65	1451	6.79	403	14.01	
2–8	11 917	49.16	10 531	49.3	1386	48.18	
9–13	1837	7.58	1659	7.77	178	6.19	
14 or more	766	3.16	665	3.11	101	3.51	
Hospital site of admission							
1	7704	31.78	6831	31.98	873	30.34	<0.001
2	12 348	50.94	10 940	51.21	1408	48.94	
3	4188	17.28	3592	16.81	596	20.72	

ICU, intensive care unit; IQR, Interquartile range.

The association between intrahospital transfers and HAI was analysed using a logistic regression model. A purposeful model selection approach was taken which considered potential confounders associated with the exposure and outcome based on previous studies and clinical opinion, alongside data-driven exploration. For each confounder included in the multivariable model, logistic regression model performance was assessed using goodness-of-fit tests and inspection of residuals to guide selection. No multicollinearity was found between independent variables. Patients with a time at risk over 57.2 days were excluded (the top 1% of time at risk distribution) as they are likely atypically complex cases with regard to procedures, treatment and comorbidities (online supplemental table 3 for results including this population). Missing data were minimal, therefore spells containing incomplete information were removed while missing microbiology spells were treated as negative. Spells containing timing inconsistencies were also removed.

Data exploration showed that patient-level clustering did not impact results (online supplemental table 4A); therefore, hospital spells from the same individual were considered independent observations, and multiple spells per patient were included if the eligibility criteria were met. In addition, TFC-level and hospital site-level clustering was found to be minimal, and conducting a multilevel logistic regression using TFC or hospital site as a second-level cluster did not meaningfully alter the results (online supplemental table 4B–D).

### Sensitivity analyses

It is possible that in some instances patients with a suspected infection are transferred to a single room on a different ward prior to sample collection. To assess the robustness of results against this, a sensitivity analysis was conducted with time at risk defined as 12 and 24 hours prior to the sample collection date. In this analysis, the case definition was accordingly updated, meaning that cases with a time at risk of less than 48 hours under the new definition were removed. A second sensitivity analysis in which surgical patients were identified for exclusion by OPCS-4 codes, as opposed to TFC, was conducted to ensure that the possibility of misclassification of medical patients under a surgical TFC does not affect the results (online supplemental tables 5A, B and 6). Finally, in our primary analysis, all positive cultures were assumed to be the first stage of an endogenous infection, allowing the possibility that some cases are misclassified due to colonisations detected in the host without causing a disease. A sensitivity analysis in which cases were restricted to positive samples isolated only from a sterile body site, defined as blood or urine, was conducted to assess the robustness of results (online supplemental table 7).

ORs with 95% CIs are reported for unadjusted and adjusted analyses, with a p value <0.05 considered to be statistically significant. Residuals were examined to confirm the normality assumption was met. All analyses were performed using STATA V.16 software (STATA, College Station, Texas) and R Studio (http://www.r-project.org).

## Results

### Patient and hospital characteristics

A total of 24 240 hospital spells were included in the analysis, pertaining to 16 018 individual patients admitted to the three hospital sites over the 3-year data collection period. Cases were defined as spells with a positive laboratory culture collected at least 48 hours after hospitalisation, while controls were defined as spells where the patients remained infection free for the entirety of their spell. The intraclass correlation coefficient (ICC) computed using hospital site as a second-level cluster was low (ICC=0.004), suggesting any differences between hospital site characteristics on rate of infection were minimal. Table 1 summarises patient characteristics by the outcome. Over the time period, 11.9% of patients developed an HAI. 49.6% of patients in the cohort were male while 50.4% were women, with a median age of 79 (IQR 72–86) and a mean of 3.5 Elixhauser comorbidities (SD 1.9).

The median time at risk was 6.3 days (IQR 3.6–11.7). General medicine (31.5%), geriatric medicine (15.1%), respiratory medicine (8.9%), cardiology (8.4%), stroke medicine (8.1%) and nephrology (5.2%) comprised 77.2% of spells. Gender and readmission within 30 days did not differ between cases and controls. However, patients who acquired an HAI were older than those who did not (median 79, IQR 73–86 vs median 79, IQR 72–85, p=0.004), and had a higher mean number of Elixhauser comorbidities (mean 4.0, SD 2.0 vs mean 3.5, SD 1.9; difference=0.5; p<0.001). Both ICU admission (6.7% vs 1.9%; difference=4.8%; p<0.001) and in-hospital death (13.3% vs 6.4%, difference=6.9%; p<0.001) were higher for cases. Significant differences were also found in proportions of procedures and time at risk intervals between cases and controls (p<0.001 and p=0.016, respectively). Table 2 describes the most frequently isolated pathogens among cases.

**Table 2:** Individual counts and percentages of the most commonly isolated pathogens comprising 81.02% of the 2877 cases are given

Organism name	n	%
*Clostridium difficile* toxin	930	32.32
*Escherichia coli*	462	16.06
*Pseudomonas aeruginosa*	250	8.69
*Enterococcus* sp	162	5.63
*Klebsiella pneumoniae*	153	5.32
*Staphylococcus aureus*	135	4.69
*Coliform* sp	99	3.44
Methicillin-resistant *Staphylococcus aureus*	73	2.54
Coagulase-negative staphylococcus	67	2.33

**Figure 2:**
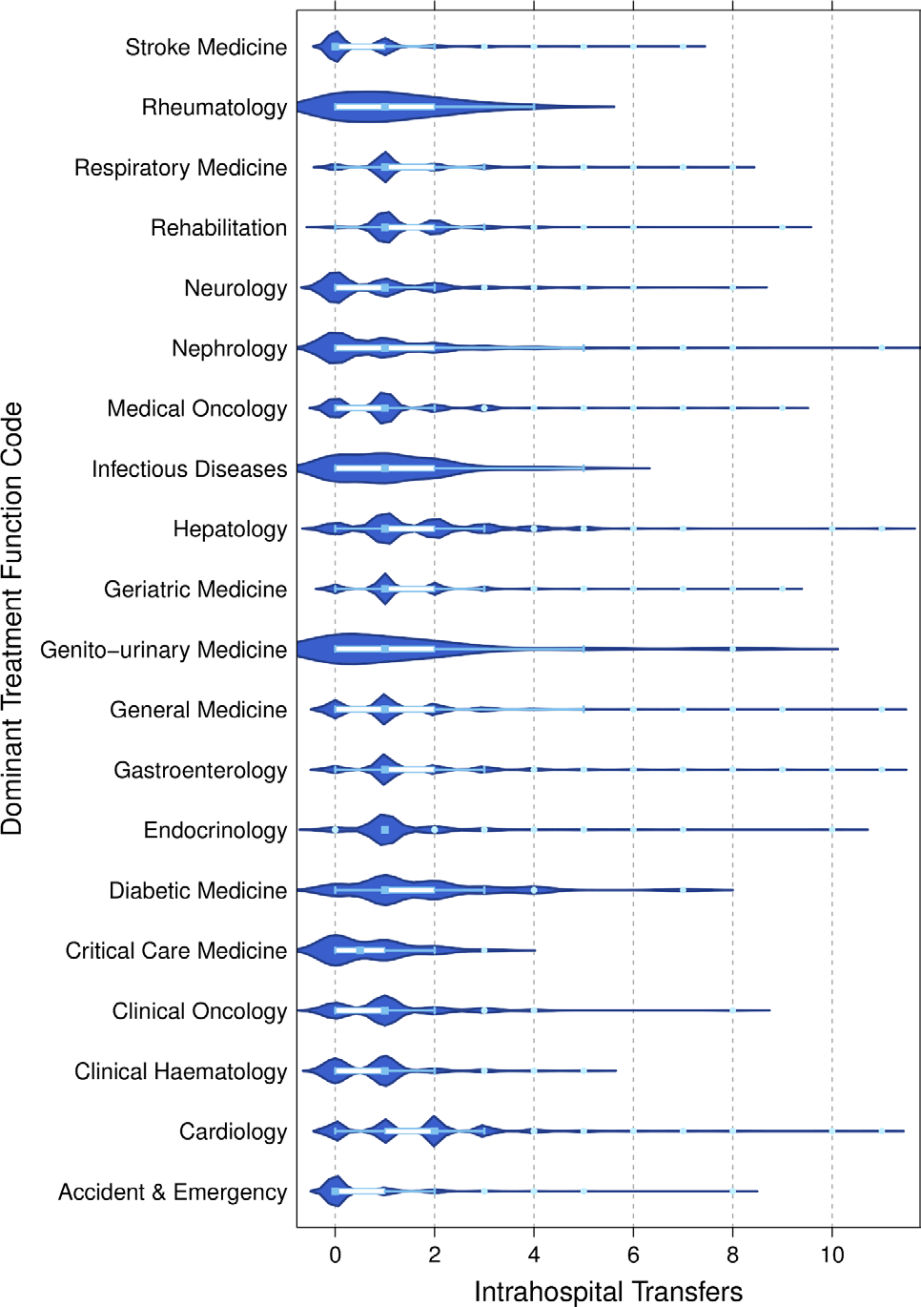
Violin and box and whisker plots of intrahospital transfers stratified by the dominant TFC the patient was listed under. The length of the box represents the IQR, the horizontal line in the box interior represents the median, the whiskers represent the 1.5 times the IQR of the 25th quartile or 1.5 times the IQR of the 75th quartile. The violin plot depicts the probability density for each TFC group at a given intrahospital transfer value.

While 27.8% of patients did not undergo any intrahospital transfers, 44.2% of patients underwent one intrahospital transfer during their spell, 17.1% underwent two transfers and 11.0% underwent three or more transfers. Cases experienced more transfers than controls with 76.0% of cases undergoing at least one transfer, compared with 71.7% of controls. Intrahospital transfers varied marginally by TFC, with cardiology patients moving most frequently (median 2, IQR 1–2). Figure 2 depicts box and whisker plots with probability densities of intrahospital transfers by TFC.

Univariable logistic regressions were used to explore the effects of possible covariates on the outcome (online supplemental table 8) and showed that ethnicity, weekend admission and readmission within 30 days were not significantly associated with the odds of developing an HAI and were excluded from the final multivariable model. Weekend admission was also modelled as an interaction in the multivariable regression model, but was not statistically significant. All other covariates included showed significant relationships, with the exception of gender, which was defined a priori as a covariate.

In the multivariable logistic regression results, it was found that each additional intrahospital transfer was associated with a 9% increase in the odds of developing an HAI (OR=1.09; 95% CI 1.05 to 1.13). Table 3 shows the results of the univariable and multivariable logistic regression analyses (full model in online supplemental table 9).

**Table 3:** Univariable and multivariable logistic regression analysis exploring the relationship between intrahospital transfers and hospital-acquired infection in 24 240 hospital spells

	OR for development of any HAI					
	Univariable model			Multivariable model*		
	OR	P value	95% CI	OR	P value	95% CI
Intrahospital transfers	1.08	<0.001	1.05 to 1.11	1.09	<0.001	1.05 to 1.13

*Multivariable model adjusted for: age, gender, time at risk, Elixhauser comorbidities, hospital site of admission, dominant treatment function code (TFC), intensive care unit (ICU) admission, number of procedures and discharge destination.

HAI, hospital-acquired infection.

### Sensitivity analysis

A similar effect estimate was seen when time at risk was defined as 12 hours prior to the collection date (OR=1.07; 95% CI 1.03 to 1.11) and 24 hours prior to the collection date (OR=1.07; 95% CI 1.02 to 11). In addition, exclusion of surgical patients by OPCS-4 code as opposed to TFC yielded an OR of 1.10 (95% CI 1.06 to 1.13). Finally, restricting positive samples only to those isolated from a sterile body site resulted in an OR of 1.11 (95% CI 1.06 to 1.16).

## Discussion

The present study demonstrates a robust association between intrahospital transfers and the development of a hospital-associated infection (HAI), with each additional transfer increasing the odds of developing an HAI by 9% in elderly patients. We believe this is the first study to examine this association using transfer data from multiple hospital sites, as well as microbiology data from more than one organism after accounting for the listed confounders. The study contributes to a small number of studies exploring this association in an analysis which considers the chronology of events, and is concordant with previous results. Our findings suggest that the decision to move a patient should be carefully considered with regard to infection risk. The use of routinely collected EHR data makes the analysis scalable, efficient and easily replicable in different settings.

The effect size is comparable to that previously reported by McHaney-Lindstrom *et al*, who used a similar time at risk approach.^15^ While the group conducted nearest neighbour matching on the admitting department to achieve a homogenous distribution of patient health conditions, procedure number can vary widely between patients in the same department, and confound the risk of the procedure with the risk of the intrahospital transfer to the procedure room. Our study used a conservative approach to adjust for interventions by including all OPCS-4 codes recorded up to infection diagnosis, and shows that these do not fully explain the odds of acquiring an infection. Cross-sectional studies have reported larger effects, with an increase of up to 59% in the odds of developing an HAI for one ward transfer compared with no transfers.^19 21^ This discrepancy likely results from the fact that cross-sectional studies do not demarcate between transfers that occurred before infection and those that occurred after infection. In a univariable analysis using intrahospital transfers for the entire hospital spell in both cases and controls, our data also showed a larger effect (OR=1.48; 95% CI 1.44 to 1.51).

The underlying hypothesis which implicates intrahospital transfers in the horizontal transmission patterns of HAIs is in line with the results from several other study designs, providing insight into possible mechanisms. Similar results have been reported with regard to the number of total roommate exposures per day and associated risk of CDI, methicillin-resistant *Staphylococcus aureus* and vancomycin-resistant *Enterococcus*.^36^ Single-patient rooms are thought to lower pathogen transmission opportunities and the incidence of HAIs through the hypothesised mechanism of reducing person-to-person contact and person-surface-person contacts.^16 37–39^As intrahospital transfers increase a patient’s exposure to both hospital surfaces and other patients, reducing non-essential transfers may be a comparable intervention, but requires less resources. In addition, a requirement of 1.7 nurses has been reported for conducting a transfer, and 1.9 nurses for receiving one.^40^ Transferred patients therefore experience extended interactions with hospital staff, which is known to promote infection spread.^18 41^ Reducing intrahospital transfers may therefore also lower opportunities for cross-infection by staff-to-patient contact. Lastly, intrahospital transfers have been shown to be a significant, and at times unaccounted for, driving factor in nursing workload.^40 42^ High workload is a known barrier to infection prevention and control practice adherence and has been shown to be a risk factor for HAI spread.^43^ The contribution of intrahospital transfers to heavy workloads may therefore increase infection transmission indirectly.

Our study has some limitations. While proxy markers were used to adjust for illness severity, the information available did not include physiological data which could be used to compute more detailed disease severity markers in order to control for patient’s baseline risk for infection. OPCS-4 codes are limited by a lack of hierarchy with regard to the invasiveness of procedures, and do not record all minor medical devices. Additionally, unavailability of information on prescription of antibiotics and proton pump inhibitors, which have been linked to increasing colonisation pressure of some pathogens, may likewise result in unobserved confounding.^44 45^ However, as we have controlled for events where a patient may be given antibiotics (procedure count and ICU admission), there is little rationale for a confounding relationship between antibiotic prescription, intrahospital transfers and HAIs. EHR information also lacks staffing levels, staff movement or casual patient movement which may be implicated in infection spread.^46–48^ It is plausible that intrahospital transfers are a marker of reduced staff capacity, but this could not be fully investigated due to lack of information on staffing. However, weekend admissions, when hospitals are typically less well staffed, were not associated with increased risk of HAIs or increased intrahospital transfers. Furthermore, in an exploratory analysis which adjusted for periods of higher admissions, the association between intrahospital transfers and HAI remained consistent (online supplemental table 10). Prevalence of HAI was higher in our sample than the previously reported English national average of 6.4%,^31^ although this may be due to the elderly population considered. There is a possibility of misclassification of colonisations as pathogenic infections, due to unavailability of symptom information. However, this non-differential classification of cases would only lower the OR towards the null (online supplemental table 3).^49^ The study is also limited by factors common to all routine data-based analyses, such as timestamp inaccuracies or diagnostic coding errors, but steps were taken to remove spells containing inconsistencies. Finally, while our findings are of relevance to elderly patients attending other National Health Service (NHS) hospitals, they may not be generalisable to a younger, less HAI susceptible population, who likely undergo fewer intrahospital transfers.

The hospital is a complex, highly connected system and intervening in one portion of the patient journey is unlikely to lead to overall improvements.^50^ Future quality improvement initiatives may include equipping low level-of-care wards with increased capacity for close monitoring in order to prevent some transfers to a higher level of care in less severely ill patients. Increasing use of portable diagnostics could also reduce transfers to procedure wards.^51 52^ In addition, hospital staff should avoid transferring infectious patients to single rooms on different wards for isolation, which may perpetuate the spread of pathogens through the hospital environment. Finally, these findings have particularly important implications for outlying patients, many of whom will be older and frailer,^8^ and necessarily experience an increase in intrahospital transfers.^53^ Admitting patients to an inappropriate ward should be balanced with the accompanying risk, and avoided in individuals who are highly susceptible to infection. This may be particularly important in light of the COVID-19 pandemic, where patient movement could increase risk of hospital-acquired COVID-19.^54^

Amid widespread bed reductions, the NHS has been accommodating more patients in fewer beds. However, an unintended consequence of this may be an increase in intrahospital transfers. The present study demonstrates that, for elderly patients, each extra intrahospital transfer confers a 9% increase in the odds of developing an HAI. Further prospective research is needed to better characterise unnecessary intrahospital transfers and consider strategies for minimising transfers. This could diminish the spread of contagious pathogens in the hospital environment and lighten workloads in a stretched healthcare system.

## Data Availability

Data may be obtained from a third party and are not publicly available. Deidentified patient data cannot be made publicly available due to information governance restrictions. Access to the data sets used in this paper via a secure environment will be reviewed on request by Imperial College Healthcare NHS Trust.
