# Supplementary material for: Association between intrahospital transfer and hospital-acquired infection in the elderly: a retrospective case–control study in a UK hospital network
Source: BMJ Qual Saf. 2021 Jan 25;30(6):457–66. doi: 10.1136/bmjqs-2020-012124 (PMC8142451; doi:10.1136/bmjqs-2020-012124)
Supplement: Supplementary data [file bmjqs-2020-012124supp001.pdf]

Supplementary Tables and Figures

Table 1: A list of the treatment function codes, from patients present in the unfiltered dataset, used to select surgical patients.

List of treatment function descriptions used to identify surgical patients	Treatment Function Code
Anaesthetics	190
Blood and Marrow Transplantation	308
Breast Surgery	103
Cardiac Surgery	172
Cardiothoracic Surgery	170
Colorectal Surgery	104
Ear Nose and Throat	120
General Surgery	100
Gynaecology	502
Hepatobiliary & Pancreatic Surgery	105
Neurosurgery	400
Ophthalmology	130
Oral Surgery	140
Paediatric Ophthalmology	216
Paediatric Surgery	171
Paediatric Trauma And Orthopaedics	214
Paediatric Urology	211
Pain Management	191
Plastic Surgery	160
Podiatric Surgery	663
Thoracic Surgery	173
Transplantation Surgery	102
Trauma & Orthopaedics	110
Urology	101
Vascular Surgery	107
Upper Gastrointestinal Surgery	106

Boncea EE, *et al. BMJ Qual Saf* 2021; 30:457–466. doi: 10.1136/bmjqs-2020-012124

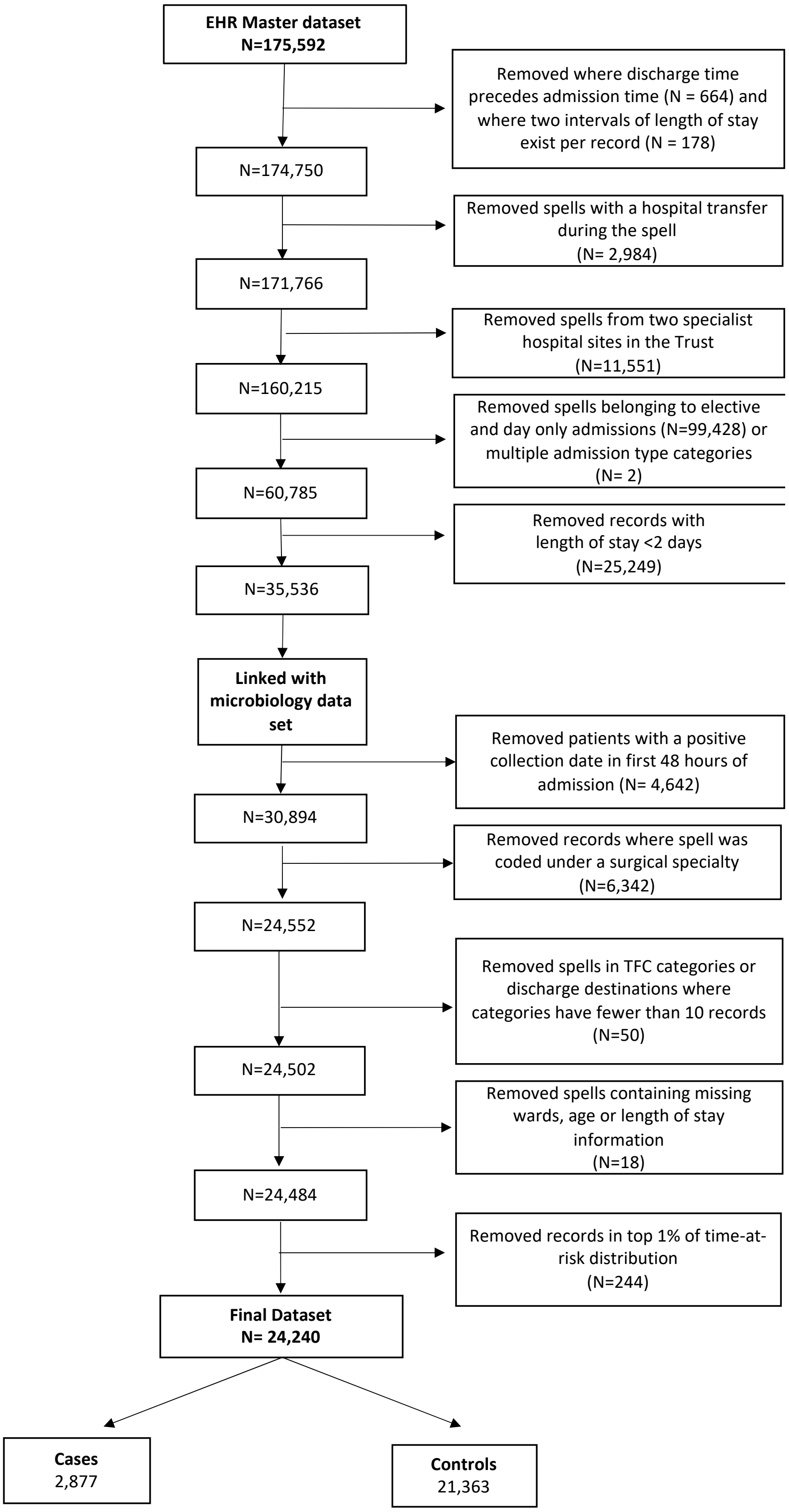

Figure 1: Flow chart depicting data linking, selection criteria and number of spells included in the analysis.

Boncea EE, *et al. BMJ Qual Saf* 2021; 30:457–466. doi: 10.1136/bmjqs-2020-012124

Continuation of Table 1 provided in main manuscript:

	All Spells (n = 24240)		Controls (n=21363)		Cases (2877)		P value
**Characteristic**	**n**	**%**	**n**	**%**	**n**	**%**	
**Treatment function code**							
Accident and Emergency	1147	4.73	1080	5.06	67	2.33	<0.001
Cardiology	2041	8.42	1823	8.53	218	7.58	
Clinical Haematology	292	1.20	227	1.06	65	2.26	
Oncology	149	0.61	133	0.62	16	0.56	
Critical Care Medicine	30	0.12	26	0.12	4	0.14	
Diabetic Medicine	33	0.14	31	0.15	2	0.07	
Endocrinology	700	2.89	546	2.56	154	5.35	
Gastroenterology	1137	4.69	993	4.65	144	5.01	
General Medicine	7642	31.53	6884	32.22	758	26.35	
Genitourinary Medicine	25	0.10	18	0.08	7	0.24	
Geriatric Medicine	3670	15.14	3177	14.87	493	17.14	
Hepatology	262	1.08	216	1.01	46	1.60	
Infectious Diseases	61	0.25	55	0.26	6	0.21	
Medical Oncology	895	3.69	812	3.8	83	2.88	
Nephrology	1256	5.18	1025	4.8	231	8.03	
Neurology	226	0.93	181	0.85	45	1.56	
Rehabilitation	526	2.17	458	2.14	68	2.36	
Respiratory Medicine	2155	8.89	1863	8.72	292	10.15	
Rheumatology	24	0.10	19	0.09	5	0.17	
Stroke Medicine	1969	8.12	1796	8.41	173	6.01	
**Discharge destination**							
Home residence	20013	82.56	17962	84.08	2051	71.29	<0.001
Temporary residence	295	1.22	257	1.20	38	1.32	
Patient died in hospital	1752	7.23	1369	6.41	383	13.31	
NHS Nursing home or Local Authority accommodation	627	2.59	500	2.34	127	4.41	
Non-NHS run care home or hospice	444	1.83	328	1.54	116	4.03	
Psychiatric unit	23	0.09	21	0.10	2	0.07	
Other NHS hospital provider	1008	4.16	861	4.03	147	5.11	
Private hospital	78	0.32	65	0.3	13	0.45	
**Readmission within 30 days***							
No	21607	89.14	19067	89.25	2540	88.29	0.118
Yes	2633	10.86	2296	10.75	337	11.71	

Boncea EE, et al. BMJ Qual Saf 2021; 30:457–466. doi: 10.1136/bmjqs-2020-012124

***in spells meeting the study eligibility criteria**

Sensitivity Analysis concerning *Clostridium difficile* infection (CDI) definition:

In our study CDI cases included patients with CDI toxin detected in a stool sample by either immunoassay or polymerase chain reaction (PCR) test (with the first result taken if both tests were performed). It was assumed that in accordance with trust guidance *C. difficile* was only investigated in patients with diarrhoeal stools, therefore our definition is in line with the European Point Prevalence Survey which states one of the criteria for a hospital-acquired *C.difficile* infection as: '*diarrhoeal stools or toxic megacolon, and a positive laboratory assay for C. difficile toxin A and/or B in stools or a toxin-producing C. difficile organism detected in stool via culture or other means, e.g. a positive PCR result…at least 48 hours after admission'.* However, as this yielded a high number of CDI cases, we conducted a sensitivity analysis in which only PCR confirmed CDI samples were included in cases. All other CDI toxin results were assumed to be false positives and reassigned to controls resulting in a total sample size of 24,851 spells (22,553 controls and 2,298 cases), and 89 positive CDI cases (comprising 3.9% of all cases). The multivariable results of the association between intrahospital transfers and hospital-acquired infection (HAI) remained consistent with the original analysis, with a small increase in OR in the univariable analysis.

Table 2: Univariable and multivariable model results in 24,851 hospital spells, with CDI cases defined by a positive PCR result.

	**Odds ratio for development of any HAI**					
	**Univariable model**			**Multivariable model***		
**Intrahospital transfers**	OR	P value	95% CI	OR	P value	95% CI
	1.10	<0.001	1.07-1.14	1.08	<0.001	1.04-1.13

*Multivariable model results were adjusted for: age, gender, time at risk, Elixhauser comorbidities, hospital site of admission, dominant treatment function code, admission to an intensive care ward, number of procedures and discharge destination.

We concluded that while CDI cases compose a large proportion of the cases, they are not influential drivers of the effect between intrahospital transfers and HAI in these data.

Boncea EE, *et al. BMJ Qual Saf* 2021; 30:457–466. doi: 10.1136/bmjqs-2020-012124

Table 3: Univariable and multivariable logistic regression analyses exploring the relationship between intrahospital transfers and hospital-acquired infection (HAI) in whole time at risk distribution of patients (n=24,484).

	**Odds ratio for development of any HAI**					
	**Univariable model**			**Multivariable model***		
**Intrahospital transfers**	OR	P value	95% CI	OR	P value	95% CI
	1.07	<0.001	1.04-1.11	1.08	<0.001	1.04-1.12

*Multivariable model results were adjusted for: age, gender, time at risk, Elixhauser comorbidities, hospital site of admission, dominant treatment function code, admission to an intensive care ward, number of procedures and discharge destination.

Text 1: Correlation between bed-transfers and ward-transfers

Unlike the ward-transfer variable in our dataset, the bed-transfer variable was not time stamped. Once a case entered the ward on which their positive sample was collected, it was not possible to tell which bed transfers happened prior to the diagnosis and which happened after the diagnosis, and possibly due to it. With this limitation in mind, we conducted an exploratory sensitivity analysis in which we included any bed changes which occurred up to the ward entry time of the ward on which a case had their sample collected (therefore likely undercounting the number of bed-transfers for cases). Additionally, the bed-transfer variable captures instances where a patient is moved to a trolley while visiting a diagnostic area, and so includes some between, as well as within ward movements. Therefore, any changes of environment including a change of ward were counted in this sensitivity analysis. Results showed that bed/inter-ward transfers were highly correlated with ward-transfers (Pearson correlation = 0.998, Spearman correlation = 0.997).

Boncea EE, *et al. BMJ Qual Saf* 2021; 30:457–466. doi: 10.1136/bmjqs-2020-012124

Alternative Model Results:

The residual intraclass correlation coefficient (ICC), which computes the proportion of variability explained by the presence of clusters was checked at the patient, treatment function code (TFC) and hospital level, and as clustering was found to be minimal a logistic regression was chosen as the final model. However, several other models were run for data exploration. Univariable and multivariable regressions were run using Stata's estimation command with the vce(cluster *clustvar)* option to obtain a robust variance estimate that adjusts for within-cluster correlation at the patient level (Table 4A). In addition, univariable and multivariable logistic multilevel models (hospital spells within TFCs, and hospital spells within hospital site) were run with both random intercepts for TFCs and hospital sites, and with random slope for intrahospital transfers (Table 4B-D), using Stata's melogit command. The model for hospital site as a second-level cluster and intrahospital transfers as a random slope did not converge. All multivariable model results were adjusted for: age, gender, time at risk, Elixhauser comorbidities, hospital site of admission, dominant treatment function code, intensive care unit admission, number of procedures and discharge destination.

Table 4A: Univariable and multivariable logistic regression results exploring the relationship between intrahospital transfers and hospital-acquired infection (HAI) using cluster-robust standard errors for clustering at the patient-level in 24,240 hospital spells corresponding to 16,018 individual patients.

	**Odds ratio for development of any HAI**					
	**Univariable model**			**Multivariable model***		
**Intrahospital transfers**	OR	P value	95% CI	OR	P value	95% CI
	1.08	<0.001	1.05-1.11	1.09	<0.001	1.05-1.13
*Multivariable model adjusted for: age, gender, time at risk, Elixhauser comorbidities, hospital site of admission, dominant treatment function code, intensive care unit admission, number of procedures and discharge destination.						

Table 4B: Results of multilevel univariable and multivariable model exploring the relationship between intrahospital transfers and hospital-acquired infection (HAI) using random intercept for treatment function code in 24,240 hospital spells.

	**Odds ratio for development of any HAI**					
	**Univariable model**			**Multivariable model***		
**Intrahospital transfers**	OR	P value	95% CI	OR	P value	95% CI
	1.06	<0.001	1.02-1.09	1.09	<0.001	1.05-1.13
*Multivariable model adjusted for: age, gender, time at risk, Elixhauser comorbidities, hospital site of admission, intensive care unit admission, number of procedures and discharge destination.						

Table 4C: Results of multilevel univariable and multivariable logistic regression exploring the relationship between intrahospital transfers and hospital-acquired infection (HAI) using a random intercept for treatment function code and random slope for intrahospital transfers in 24,240 hospital spells.

	**Odds ratio for development of any HAI**					
	**Univariable model**			**Multivariable model**		
**Intrahospital transfers**	OR	P value	95% CI	OR	P value	95% CI
	1.06	0.045	1.00-1.12	1.08	0.003	1.02-1.13

*Multivariable model adjusted for: age, gender, time at risk, Elixhauser comorbidities, hospital site of admission, intensive care unit admission, number of procedures and discharge destination.

Table 4D: Results of multilevel univariable and multivariable exploring the relationship between intrahospital transfers and hospital-acquired infection (HAI) using a random intercept for hospital site of admission in 24,240 hospital spells.

	**Odds ratio for development of any HAI**					
	**Univariable model**			**Multivariable model**		
**Intrahospital transfers**	OR	P value	95% CI	OR	P value	95% CI
	1.08	<0.001	1.04-1.11	1.08	<0.001	1.04-1.12

*Multivariable model adjusted for: age, gender, time at risk, Elixhauser comorbidities, dominant treatment function code, intensive care unit admission, number of procedures and discharge destination.

Sensitivity analysis using time at risk prior to the collection date:

Table 5A: Univariable and multivariable model exploring the relationship between intrahospital transfers and hospital-acquired infection (HAI) when specifying time at risk as 12 hours prior to a positive sample collection (n=24,012).

	**Odds ratio for development of any HAI**					
	**Univariable model**			**Multivariable model***		
**Intrahospital transfers**	OR	P value	95% CI	OR	P value	95% CI
	1.07	<0.001	1.04-1.11	1.07	0.001	1.03-1.11

*Multivariable model adjusted for: age, gender, time at risk, Elixhauser comorbidities, hospital site of admission, dominant treatment function code, intensive care unit admission, number of procedures and discharge destination.

Boncea EE, *et al. BMJ Qual Saf* 2021; 30:457–466. doi: 10.1136/bmjqs-2020-012124

Table 5B: Univariable and multivariable model exploring the relationship between intrahospital transfers and hospital-acquired infection (HAI) when specifying time at risk as 24 hours prior to sample collection date for patients (n=23,781).

	Odds ratio for development of any HAI					
	Univariable model			Multivariable model		
**Intrahospital transfers**	OR	P value	95% CI	OR	P value	95% CI
	1.07	<0.001	1.04-1.11	1.07	0.002	1.02 -1.11

*Multivariable model adjusted for: age, gender, time at risk, Elixhauser comorbidities, hospital site of admission, dominant treatment function code, intensive care unit admission, number of procedures and discharge destination.

Sensitivity analysis for surgical patient selection strategy by OPCS-4 codes.

While Office of Population Censuses and Surveys Classification of Interventions and Procedures (OPCS-4) intervention codes capture a wide range of procedures, they do not weigh majorly invasive procedures differently to minimally invasive procedures. They are broadly categorised into major, intermediate, minor and non-operative procedures, but the individual codes themselves are not classified. The national clinical coding standard does however provide some key words associated with each of these groups. Therefore, the OPCS-4 code descriptions were searched for the terms "Total removal", "Replacement", "Transplant", "Partial removal", "Destruction" and "Reconstruction Repair" which are associated with major and intermediate procedures. This returned 1,250 individual codes. Spells which contained these codes at any point were removed from this analysis (n=1091) resulting in a final patient sample of 29,427.

Table 6: Univariable and multivariable logistic regression analysis exploring the relationship between intrahospital transfers and hospital-acquired infection (HAI) in medical patients ascertained by OPCS-4 codes.

	Odds ratio for development of any HAI					
	Univariable model			Multivariable model		
**Intrahospital transfers**	OR	P value	95% CI	OR	P value	95% CI
	1.09	<0.001	1.06-1.12	1.10	<0.001	1.06-1.13

*Multivariable model adjusted for: age, gender, time at risk, Elixhauser comorbidities, hospital site of admission, dominant treatment function code, intensive care unit admission, number of procedures and discharge destination.

Sensitivity analysis using only positive samples collected from sterile sites:

In order to address the potential for misclassification due to colonisations in all types of pathogens, we conducted a sensitivity analysis in which we restricted cases to positive

samples collected from a sterile site. A sterile site included any pathogens identified from a blood or urine sample. Urine from catheters and nephrostomy bags were included, however samples associated with all other devices were excluded. Patients with an infection identified from any other source were assumed to be colonisations or contaminations, rather than true pathogenic infections, and allocated to the control group. This necessarily excluded any infections detected by targeted samples (such as CDI infections which are identified from stool samples) from the case count, which may have resulted in some controls being misclassified. The site of the sample collection in our data was a free text variable, giving over 2000 unique sites. We firstly filtered for blood and urine, and then excluded any sites that appeared to be associated with an indwelling device (e.g. blood line), or a wound/clot. We also note that it was assumed aseptic technique was performed during the sample collection, and a limitation of this analysis is that this cannot be confirmed. Results showed increase in the observed OR from 1.09 (95%CI 1.05 to 1.13) in the primary analysis to 1.11 (95% CI 1.06 to 1.16) (Table 7). Although this estimate is less precise it suggests that some cases in our main analysis are misclassified colonisations, and therefore nondifferential misclassification of the outcome has occurred (as this misclassification does not depend on the main exposure) resulting in a lower OR.

Table 7: Univariable and multivariable logistic regression analysis exploring the relationship between intrahospital transfers and any hospital-acquired infection (HAI) diagnosed through blood or urine cultures in 26,594 hospital spells.

	**Odds ratio for development of any HAI**					
	**Univariable model**			**Multivariable model***		
**Intrahospital transfers**	OR	P value	95% CI	OR	P value	95% CI
	1.13	<0.001	1.09-1.17	1.11	<0.001	1.06-1.16

*Multivariable model adjusted for: age, gender, time at risk, Elixhauser comorbidities, hospital site of admission, dominant treatment function code, intensive care unit admission, number of procedures and discharge destination.

Univariable Results

Table 8: Univariable logistic regression analyses exploring the relationship between covariates and outcome of hospital acquired infection.

	**Odds Ratio**	**P value**	**Lower 95% CI**	**Upper 95%CI**
**Gender**				
Male	*Reference*			
Female	0.98	0.64	0.91	1.06
**Age**				

65-70	*Reference*			
71-75	1.15	0.04	1.01	1.32
76-80	1.21	0.004	1.06	1.37
81-85	1.17	0.02	1.03	1.33
over 86	1.26	<0.001	1.12	1.42
**Attended ICU**				
No	*Reference*			
Yes	3.72	<0.001	3.12	4.44
**Time at risk (days)**				
2-5 days	*Reference*			
5-7 days	1.10	0.12	0.98	1.23
7-10 days	0.93	0.25	0.82	1.05
10-15	0.99	0.85	0.87	1.12
15-20	1.05	0.52	0.90	1.24
20-30	1.21	0.02	1.03	1.42
30-40	0.97	0.83	0.75	1.26
Over 40	0.63	0.02	0.43	0.93
**Elixhauser comorbidities**				
0	*Reference*			
1-3	1.61	0.003	1.18	2.20
4-6	2.37	<0.001	1.73	3.23
7-9	3.19	<0.001	2.29	4.44
10 or more	4.37	<0.001	2.38	8.02
**Procedures**				
No procedures	*Reference*			
1	2.42	<0.001	2.12	2.77
2-8	1.15	0.003	1.05	1.26
9-13	0.94	0.45	0.79	1.11
14 or more	1.32	0.01	1.06	1.65
**Hospital site of admission**				
Hospital Site 1	*Reference*			
Hospital Site 2	1.01	0.88	0.92	1.10
Hospital Site 3	1.30	<0.001	1.16	1.45
**Dominant treatment function code**				
Accident & Emergency	*Reference*			
Cardiology	1.93	<0.001	1.45	2.56
Clinical Haematology	4.62	<0.001	3.19	6.68
Clinical Oncology	1.94	0.02	1.09	3.44
Critical Care Medicine	2.48	0.10	0.84	7.31
Diabetic Medicine	1.04	0.96	0.24	4.44
Endocrinology	4.55	<0.001	3.35	6.17
Gastroenterology	2.34	<0.001	1.73	3.16
General Medicine	1.77	<0.001	1.37	2.30

Genitourinary Medicine	6.27	<0.001	2.53	15.53
Geriatric Medicine	2.50	<0.001	1.92	3.26
Hepatology	3.43	<0.001	2.29	5.14
Infectious Diseases	1.76	0.21	0.73	4.23
Medical Oncology	1.65	0.003	1.18	2.30
Nephrology	3.63	<0.001	2.73	4.83
Neurology	4.01	<0.001	2.66	6.03
Rehabilitation	2.39	<0.001	1.68	3.41
Respiratory Medicine	2.53	<0.001	1.92	3.33
Rheumatology	4.24	0.01	1.54	11.71
Stroke Medicine	1.55	<0.001	1.16	2.08
**Discharge destination**				
Home residence	*Reference*			
Temporary residence	1.29	0.14	0.92	1.83
Patient died in hospital	2.45	<0.001	2.17	2.77
NHS Nursing home or Local Authority accommodation	2.22	<0.001	1.82	2.72
Non-NHS run care home or hospice	3.10	<0.001	2.49	3.85
Psychiatric unit	0.83	0.81	0.20	3.56
Other NHS hospital provider	1.50	<0.001	1.25	1.79
Private hospital	1.75	0.07	0.96	3.18
**Ethnic code description**				
African	*Reference*			
Any other Asian background	0.94	0.73	0.68	1.31
Any other Black background	0.76	0.22	0.49	1.17
Any other White background	0.89	0.44	0.67	1.19
Any other ethnic group	0.92	0.54	0.69	1.21
Any other mixed background	0.71	0.35	0.36	1.44
Bangladeshi	0.91	0.74	0.51	1.62
British	0.99	0.96	0.77	1.29
Caribbean	0.98	0.89	0.72	1.32
Chinese	0.70	0.32	0.35	1.41
Indian	1.29	0.10	0.95	1.75
Irish	1.02	0.89	0.76	1.38
Not known	1.06	0.80	0.69	1.62
Not stated	0.83	0.20	0.62	1.10
Pakistani	0.97	0.90	0.61	1.55
White and Asian	0.36	0.16	0.08	1.51
White and Black African	*Empty*			

Boncea EE, *et al. BMJ Qual Saf* 2021; 30:457–466. doi: 10.1136/bmjqs-2020-012124

White and Black Caribbean	1.21	0.59	0.61	2.41
**Weekend admission**				
Weekday admission	*Reference*			
Weekend admission	0.95	0.32	0.87	1.04
**Readmission within 30 days***				
No	*Reference*			
Yes	1.10	0.118	0.98	1.24

***in spells meeting the study eligibility criteria**

Multivariable results:

Table 9: Full multivariable logistic regression analysis exploring the relationship between independent variables and outcome of hospital acquired infection. Multivariable model results were adjusted for: age, gender, time at risk, Elixhauser comorbidities, hospital of admission, dominant treatment function code (TFC), admission to intensive care unit (ICU), number of procedures and discharge destination.

	**Odds Ratio**	**P value**	**Upper 95%CI**	**Lower 95% CI**
**Intrahospital transfers**	1.09	<0.001	1.05	1.13
**Gender**				
Male	*Reference*			
Female	1.01	0.73	0.93	1.10
**Age**				
65-70	*Reference*			
71-75	1.19	0.01	1.04	1.37
76-80	1.27	0.001	1.11	1.45
81-85	1.29	<0.001	1.12	1.48
Over 86	1.41	<0.001	1.23	1.61
**Attended ICU**				
No	*Reference*			
Yes	3.56	<0.001	2.91	4.35
**Time at risk (days)**				
2-5	*Reference*			
5-7	0.87	0.02	0.77	0.98
7-10	0.65	<0.001	0.57	0.74
10-15	0.61	<0.001	0.53	0.70
15-20	0.56	<0.001	0.47	0.67
20-30	0.56	<0.001	0.47	0.68
30-40	0.41	<0.001	0.31	0.55
Over 40	0.22	<0.001	0.14	0.33
**Elixhauser comorbidities**				
0	*Reference*			
1-3	1.55	0.01	1.13	2.13
4-6	2.15	<0.001	1.56	2.96

Boncea EE, *et al. BMJ Qual Saf* 2021; 30:457–466. doi: 10.1136/bmjqs-2020-012124

7-9	2.81	<0.001	2.00	3.94
10 or more	2.80	0.002	1.47	5.35
**Procedures**				
No procedures	*Reference*			
1	2.27	<0.001	1.98	2.61
2-8	1.10	0.05	1.00	1.22
9-13	0.83	0.05	0.69	1.00
14 or more	0.99	0.92	0.77	1.27
**Hospital site**				
Hospital Site 1	*Reference*			
Hospital Site 2	1.06	0.31	0.95	1.17
Hospital Site 3	1.36	0.01	1.07	1.74
**Dominant TFC**				
Accident and Emergency	*Reference*			
Cardiology	1.09	0.64	0.75	1.59
Clinical Haematology	4.07	<0.001	2.60	6.35
Clinical Oncology	2.02	0.02	1.12	3.65
Critical Care Medicine	0.77	0.65	0.25	2.37
Diabetic Medicine	1.08	0.92	0.25	4.71
Endocrinology	4.53	<0.001	3.30	6.22
Gastroenterology	2.26	<0.001	1.65	3.10
General Medicine	1.46	0.01	1.12	1.90
Genitourinary Medicine	7.58	<0.001	3.00	19.18
Geriatric Medicine	2.39	<0.001	1.82	3.15
Hepatology	3.50	<0.001	2.30	5.32
Infectious Diseases	1.34	0.53	0.54	3.29
Medical Oncology	1.55	0.01	1.10	2.20
Nephrology	2.96	<0.001	2.03	4.32
Neurology	3.84	<0.001	2.49	5.93
Rehabilitation	2.53	<0.001	1.74	3.67
Respiratory Medicine	2.40	<0.001	1.81	3.19
Rheumatology	3.56	0.02	1.23	10.34
Stroke Medicine	1.29	0.11	0.95	1.76
**Discharge location**				
Home residence	*Reference*			
Temporary residence	1.42	0.05	1.00	2.02
Patient died in hospital	2.09	<0.001	1.83	2.38
NHS Nursing home or Local Authority accommodation	2.89	<0.001	2.33	3.58
Non-NHS run care home or hospice	3.73	<0.001	2.96	4.69
Psychiatric unit	1.09	0.90	0.25	4.75
Other NHS hospital	1.88	<0.001	1.53	2.31

provider				
Private hospital	2.15	0.02	1.15	4.01

Exploratory covariate for periods of higher admissions:

Seasonal effects which correlate with higher numbers of hospital-acquired infections (HAIs) would ideally have been explored as a covariate, but we were unable account for seasonality in order to respect the terms set by the Data Protection Office, which stipulated that dates of patient admission remain de-identified.

An exploratory analysis was conducted in which admission dates covering the 36-months of admissions were allotted into four quarters of consecutive three-month intervals for each year, creating a categorical variable. We produced three such variables, the first of which began from Day 0 in the data, the second from day 30 and the third day 60, in order to account for the possibility that the data collection began mid-quarter. We then chose the variable with the largest difference in admissions between the smallest and largest quarter, which therefore best discriminated between 3-month intervals with higher and lower number of admissions (Table 10A). As influxes in admissions of any kind, not only the patients in our final cohort, would have contributed to resource strain we used everyone available in the dataset for this analysis (with the exclusion of some erroneous spells and those that belonged to the specialist hospitals).

Table 10A: Number of hospital admissions by quarters of 12 months (n = 531,491). While admissions remain relatively constant, an increase in admissions is observed in Q3 and Q4.

	Frequency	*Percentage*	*Cumulative*
Q1	129114	24.29	24.29
Q2	131234	24.69	48.98
Q3	134672	25.34	74.32
Q4	136471	25.68	100

This variable was found to be a significant predictor of HAI development, but it did not impact the association between intrahospital transfers and HAI development (Table 10B).

Table 10B: Univariable and multivariable logistic regression analysis exploring the relationship between intrahospital transfers and hospital-acquired infections (HAI) in 24,240 hospital spells. The admissions flag category with least cases was used as the reference.

	**Odds ratio for development of any HAI**					
	**Univariable model**			**Multivariable model***		
	OR	P value	95% CI	OR	P value	95% CI
Intrahospital transfers	1.08	<0.001	1.05-1.12	1.09	<0.001	1.05-1.13
Admission flag						
Q1	1.82	<0.001	1.62-2.04	1.93	<0.001	1.72-2.17
Q2	1.22	<0.001	1.08-1.38	1.28	<0.001	1.12-1.45
Q3	reference					

Q4	1.62	<0.001	1.44-1.82	1.66	<0.001	1.47-1.87

*In addition to the admissions flag, multivariable was adjusted for: age, gender, time at risk, Elixhauser comorbidities, hospital site of admission, dominant treatment function code, intensive care unit admission, number of procedures and discharge destination.

Boncea EE, *et al. BMJ Qual Saf* 2021; 30:457–466. doi: 10.1136/bmjqs-2020-012124
